# Ultrasound treatment inhibits browning and improves antioxidant capacity of fresh-cut sweet potato during cold storage

**DOI:** 10.1039/c9ra06418d

**Published:** 2020-03-03

**Authors:** Yanfang Pan, Lan Chen, Lingling Pang, Xiaotong Chen, Xiaoyu Jia, Xihong Li

**Affiliations:** Key Laboratory of Food Nutrition and Safety, Ministry of Education, Tianjin University of Science and Technology Tianjin 300457 China lixihong@tust.edu.cn; Tianjin Gasin-DH Preservation Technology Co., Ltd Tianjin 300300 China

## Abstract

Controlling browning and mitigating oxidative damage are important factors when attempting to extend the shelf-life and high-quality features of fresh-cut sweet potato (*Ipomoea batatas* (L.) Lam). In order to preserve the color and antioxidant capacity, ultrasound (US) treatment at 40 kHz for 10 min was applied to investigate the effect on enzymatic browning of sweet potato slices. Changes in color, total phenolic content, total antioxidant capacity, phenol metabolism-related enzymes including phenylalanine ammonia-lyase (PAL), polyphenol oxidase (PPO), peroxidase (POD) were examined. Also investigated here were superoxide radical (O_2_^−^˙) and hydrogen peroxide (H_2_O_2_) contents, antioxidant enzymes including superoxide dismutase (SOD) and catalase (CAT) involved in reactive oxygen species metabolism. After storage lasting 10 days at 4 °C, US-treated slices maintained significantly (*p* < 0.05) higher luminosity (*p* = 0.000003) and chroma (*p* = 0.000018) by reducing PPO and POD activities, when compared to the control. Meanwhile, the induction of PAL was observed to positively correlate with higher total phenolic content (*r* = 0.818, *p* < 0.01; *p* = 6.1752 × 10^−9^), thereby enhancing antioxidant capacity to combat oxidative damage. Moreover, O_2_^−^˙ (*p* = 3.8046 × 10^−10^) and H_2_O_2_ (*p* = 0.000013) concentrations were significantly (*p* < 0.05) suppressed by activating CAT and SOD activities. Results suggested that US treatment could inhibit browning of fresh-cut sweet potato by reducing the activity of PPO and POD while improving total antioxidant capacity.

## Abstract 2

Ultrasound treatment could inhibit browning of fresh-cut sweet potato by reducing PPO and POD activities while improving total antioxidant capacity.

## Graphical abstract

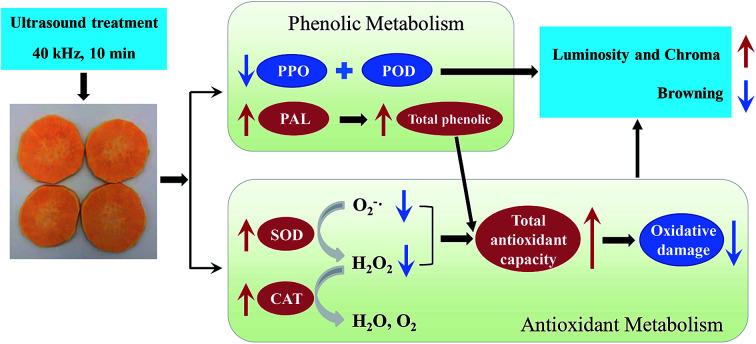

## 1. Introduction

As the world's seventh most important food crop, sweet potato (*Ipomoea batatas* (L.) Lam) is widely grown in more than 100 countries and regions.^1^ Its tuberous root is a good source for food processing with high levels of carbohydrates and vitamins and possesses strong antioxidant potential.^2,3^ With the demands of modern society's fast-paced lifestyle, fresh-cut and ready-to-eat produce represent a market trend and have increased more than 30% in the past decade. This is due to its convenience, high utilization and nutrition retention.^4,5^ Fresh-cut sweet potato is a new type of processed product for consumers or for the catering industry, and the appearance and nutritional benefits are the main factors affecting consumers' purchase choices.^6^ However, fresh-cut products are susceptible to physiological changes resulting from mechanical operations, which lead to browning discoloration, phenolic oxidative degradation, reactive oxygen metabolism maladjustment, which may eventually increase perishability and shorten shelf-life.^7–9^

It is widely believed that enzymatic browning is mainly caused by the oxidation reaction in which polyphenol oxidase (PPO) or peroxidase (POD) catalyzes the endogenous phenolic substances into quinones under aerobic conditions, and oxidize and polymerize to dark colored pigments thereafter.^10^ Phenylalanine ammonia-lyase (PAL) is considered to be another enzyme closely related to browning by transforming l-phenylalanine into phenolic substances to provide the substrate for enzymatic browning.^11^ Fresh-cut processing can cause the loss of tissue structure and cell integrity of sweet potato, which affects the distribution of phenol and phenolase, resulting in contact being made between enzymes and substrates and inducing the enzymatic browning reaction.^12^ Meanwhile, mechanical damage caused by cutting can also induce the production of large amounts of reactive oxygen species (ROS) including superoxide radical (O_2_^−^˙) and hydrogen peroxide (H_2_O_2_), and excessive ROS will aggravate membrane lipid peroxidation, cause damage to membrane integrity, thus accelerating the occurrence of enzymatic reactions.^13–15^ Therefore, regulating related enzyme activities and improving antioxidant capacity to alleviate oxidative damage induced by ROS can effectively control the browning of fresh-cut sweet potato during the storage.

Post-processing technologies have focused on the use of various anti-browning agents such as the use of chitosan, ascorbic acid, sodium hypochlorite, and so on to prevent browning of fresh-cut sweet potato.^16–18^ However, adding chemical reagents may affect the taste and consumers' acceptance of the final product. Compared with the toxic side effects of chemical methods, physical anti-browning technologies have been widely used because they are safe and effective methods. Among them, ultrasound (US) application has received increasing attention due to its effectiveness despite of being a non-thermal treatment and free-mechanical damage.^19,20^ The inhibition of browning by US is mainly due to the inactivation of enzymes caused by physical and chemical cavitation.^21^ On the one hand, high energy free radicals generated by cavitation may change the spatial conformation and biological activity of enzyme by reacting with some amino acid residues.^22^ On the other hand, shock wave and shear force produced by the collapsed of cavitation bubbles can disrupt the hydrogen bonds and van der Waals interaction in the polypeptide chain, resulting in modification of the secondary and tertiary structures of enzyme molecules.^22,23^ US has been shown to inhibit enzymatic browning and maintain fruit quality in fresh-cut strawberry,^24^ fresh-cut potato,^23^ and fresh-cut apple.^25^ Also, US proved to be effective in reducing malondialdehyde content and maintaining cell wall integrity in fresh-cut cucumber,^26^ and preserving antioxidants and extending the shelf-life of white mushroom.^27^ In one particular study, Yeoh and Ali^28^ reported that fresh-cut pineapple treated with US showed an increase in PAL activity and total phenolic content and improved antioxidant capacity in cold storage conditions. In general, US produced a variety of positive effects on anti-browning and occupied an important position in postharvest fruit processing. So far, studies on US treatment of fresh-cut sweet potato are limited, especially with reference to enzymatic browning, ROS metabolism, antioxidant activity and how these factors interrelated.

Therefore, the main objective of this analysis was to investigate indicators related to browning and antioxidant metabolism of fresh-cut sweet potato in response to US, and stored at 4 °C for 10 days. The color, total phenolic content and related metabolism enzymes (PPO, POD and PAL) were investigated, as well as ROS metabolism (O_2_^−^˙ and H_2_O_2_ concentration), related antioxidant enzymes including superoxide dismutase (SOD) and catalase (CAT) and antioxidant capacity were analyzed during the extended refrigerated storage, in order to develop a potential strategy to: firstly, control browning; and secondly, mitigate oxidative damage in fresh-cut sweet potato processing.

## 2. Materials and methods

### 2.1. Material

Sweet potato tuberous roots of cultivar ‘Longshu No. 9’ were obtained from a plantation located in Xiongxian County, Hebei Province, China, and transported to the laboratory on the same day. Roots that were disease-free with uniform size and shape (weighing about 350–400 g), and indicating no mechanical damage were selected for fresh-cut slices processing. Sweet potato roots were flushed under tap water for 1 min, air-dried at room temperature (25 °C), and then peeled and cut into slices with a thickness of 4–5 mm. Peeler, knife and cutting board were disinfected with 0.1% (v/v) sodium hypochlorite solution before usage. A total of 300 sweet potato slices were obtained after 2 h of processing, then all slices were mixed and randomly allocated into two groups of 150 slices each for subsequent treatment.

### 2.2. Ultrasound treatment

Ultrasound treatment was conducted in 230 mm × 140 mm × 100 mm (length × width × height) ultrasound bath with a KQ2200DE ultrasound apparatus (Kunshan Ultrasonic Instrument Co., Ltd, China), operating at 40 kHz frequency and 100 W power density for 10 min at 25 °C. This ultrasound exposure time was chosen as the best one based on preliminary experiments for 5, 10, 15 and 20 min, and resulted in significant reduction of spoilage microorganisms enumerated from sweet potato slices. Fresh-cut sweet potatoes immersed in distilled water for 10 min at 25 °C served as the control. Subsequently, all treated slices were drained by gauze, then packed into polyethylene bags (280 mm × 180 mm) and stored at 4 °C for 10 d. All treatments were replicated three times and changes in relevant indicators were measured every 2 d.

### 2.3. Color evaluation

The color of fresh-cut sweet potato was determined using a HP-200 automatic colorimeter (Shanghai Hanpu Photoelectric Technology Co., Ltd, China). Three points were randomly selected from each side of all slices for measurement (6 times for each sample). Numerical values of L* (light/dark), a* (red/green) and b* (yellow/blue) were recorded, where L* value represents luminosity, while a* and b* were converted into chroma (C*) according to the equation: C* = (a*^2^ + b*^2^)^1/2^.^29^

### 2.4. Total phenolic content determination

The total phenolic content was determined using Folin–Ciocalteu method as described by Liu *et al.*^30^ with some modifications. Briefly, ten grams of the fresh sweet potato sample were ground into liquid nitrogen, then the powder was added with 10.0 mL of 80% (v/v) acetone and centrifuged at 10 000 × *g* for 10 min. After that, 1.0 mL supernatant was added with 2.0 mL of Folin-phenol reagent and 10.0 mL of 10% (w/w) sodium carbonate solution, shake well and placed in a dark place for 60 min. The absorbance value at 765 nm was measured using an UV-spectrophotometer (Shimadzu UV-1800, Japan) and total phenolic content was expressed as chlorogenic acid equivalent (CAE) values in mg g^−1^ fresh weight (FW).

### 2.5. Phenolic metabolism enzymes analysis

Crude enzyme extraction of PPO and POD was performed at 4 °C by a previous method with slight modifications.^30^ Tissue (5 g) from triplicate samples was homogenized in 5 mL of 0.1 mol L^−1^ sodium acetate buffer (pH 5.5) containing 1 mM polyethylene glycol 6000, 4% (w/v) polyvinylpolypyrrolidone and 1% (v/v) Triton X-100. After centrifugation at 10 000 × *g* for 15 min, the supernatant was used for enzyme assay.

PPO activity was analyzed according to the method described by Zhou *et al.*^31^ with slight modifications. The crude enzyme (1.0 mL) was reacted with 3.9 mL of 100 mM sodium phosphate buffer (pH 6.5) and 1.0 mL of 100 mM catechol. Immediately after, the absorbance at 420 nm (*A*_420_) was recorded every 30 s for 3 min. PPO activity was defined as the change of 0.01 in *A*_420_ per g per min and expressed as U g^−1^ FW.

POD activity was analyzed according to the method used by Xu *et al.*^32^ with slight modifications. The crude enzyme (1.0 mL) was reacted with 6.0 mL of 25 mM sodium phosphate buffer (pH 7.8), 2.0 mL of 0.5 M guaiacol and 1.0 mL of 2% H_2_O_2_. Absorbance at 470 nm (*A*_470_) was measured every 1 min for a total of 6 times. POD activity was defined as a change of 0.01 in *A*_470_ per g per min and expressed as U g^−1^ FW.

PAL activity was measured according to the previous method with some modifications.^33^ About 2 g of sweet potato slices were homogenized with 5 mL of boric acid buffer (pH 8.8) containing 40 g L^−1^ PVP, 2 mM EDTA and 5 mM β-mercaptoethanol in an ice bath. The homogenate was centrifuged at 5000 × *g* for 25 min at 4 °C and the supernatant was collected as crude enzyme extract. PAL activity was measured by incubating 1 mL enzyme extract at 37 °C for 60 min with 5 mL boric acid buffer mentioned above and 1 mL of 20 mM l-phenylalanine solution. The substrate was added after 10 min of preincubation and the reaction ceased with 0.1 mL of 6 N HCl. Finally, the increase in absorbance at 290 nm (*A*_290_) was measured. PAL activity was defined as a change of 0.01 in *A*_290_ per g per h and expressed as U g^−1^ FW.

### 2.6. O_2_^−^˙ and H_2_O_2_ concentrations measurement

O_2_^−^˙ was measured using the method of Xu *et al.*^34^ with slight modifications. First, 5 g of fresh sweet potato sample was homogenized in 5.0 mL of 50 mM sodium phosphate buffer (pH 7.8) and centrifuged at 12 000 × *g* for 20 min at 4 °C. Next, 1.0 mL of supernatant was mixed with 1.0 mL of 50 mM sodium phosphate buffer (pH 7.8) and 1.0 mL of 1 mM hydroxylamine hydrochloride. After incubation at 25 °C for 1 h, 1.0 mL of 17 mM *p*-aminobenzene sulfonic acid and 1.0 mL of α-naphthylamine were added to the incubation mixture; the mixture was incubated for a further 20 min for color reaction. Finally, the absorbance at 530 nm was measured and O_2_^−^˙ concentration was expressed as μmol g^−1^ FW.

H_2_O_2_ was extracted by homogenizing fresh tissue (5 g) with 5.0 mL of cold acetone and centrifuged at 12 000 × *g* for 20 min at 4 °C. Its concentration was measured according to the method employed by Patterson *et al.*^35^ with slight modifications. It involved incubating 1.0 mL extracted supernatant with 0.1 mL of 10% titanium tetrachloride–hydrochloric acid and 0.2 mL of concentrated NH_4_OH and centrifuging at 12 000 × *g* for 15 min. Then the sediment was dissolved in 3.0 mL of 2 M H_2_SO_4_ and the absorbance at 412 nm was measured. H_2_O_2_ concentration was expressed as μmol g^−1^ FW.

### 2.7. Extraction and assay of SOD and CAT

For enzyme extraction, tuberous root slices (4 g) were homogenized in 5 mL of 50 mM sodium phosphate buffer (pH 7.8) containing 5 mM dithiothreitol (DTT) and 5% polyvinylpyrrolidone (PVP) in an ice bath. The homogenates were centrifuged at 12 000 × *g* for 30 min at 4 °C and the supernatant was collected for determining SOD and CAT enzyme activity.

SOD activity was assayed utilizing Vicente *et al.*^36^ method with some modifications. The reaction mixture consisted of 1.7 mL of 50 mM sodium phosphate buffer (pH 7.8), 0.3 mL of 130 mM methionine, 0.3 mL of 750 μM nitro blue tetrazolium, 0.3 mL of 20 μM riboflavin, 0.3 mL of 0.1 mM EDTA and 0.1 mL of enzyme extract. The mixture was homogenized and placed under a 30 W fluorescent lamp for 15 min. After that, the reaction ceased immediately in a dark place. Absorbance at 560 nm was measured while the unilluminated tube served as the blank control. One unit of SOD activity was defined as the inhibition of the photo oxidation reduction by 50% per minute per gram and expressed as U g^−1^ FW.

CAT activity was measured with the method employed by Bassal and El-Hamahmy^37^ with slight modifications, by incubating 0.1 mL enzyme extract with 2.9 mL of 20 mM H_2_O_2_ at 25 °C. Absorbance at 240 nm was measured in 30 s intervals for 5 min with distilled water as the blank reference. One unit of CAT activity was defined as a change of 0.01 in *A*_240_ per minute per gram and expressed as U g^−1^ FW.

### 2.8. Determination of total antioxidant capacity

The total antioxidant activity was evaluated using DPPH (2,2-diphenyl-1-picrylhydrazyl) assay with the method of Brand-Williams *et al.*^38^ with some modification. Sweet potato slices (2 g) were homogenized in 50 mL of 70% ethanol and then sonicated for 30 min. After being centrifuged at 12 000 × *g* for 30 min at 4 °C, the supernatant was collected for determining antioxidant capacity. The reactive mixtures included 0.3 mL of supernatant and 2.7 mL of 0.2 mM DPPH in methanol solution and 0.09 mL of distilled water. Absorbance at 517 nm was measured against the blank after reaction in the dark for 30 min. Antioxidant capacity was calculated by the following formula: DPPH scavenging capacity (%) = [(*A*_0_ − *A*_i_)/*A*_0_] × 100, where *A*_0_ and *A*_i_ represent the absorbance of the control and the sample, respectively.

### 2.9. Statistical analysis

All experimental data were obtained from three replications for each treatment. Data values were expressed as mean ± standard errors. The SPSS (SPSS_19.0 for Windows) statistical software was used to calculate the analysis of variance (ANOVA). Significant differences were determined at the *p* < 0.05 level. The graphics software program used to create the figures was Origin 8.

## 3. Results and discussion

### 3.1. Color changes in sweet potato slices

**Fig. 1:**
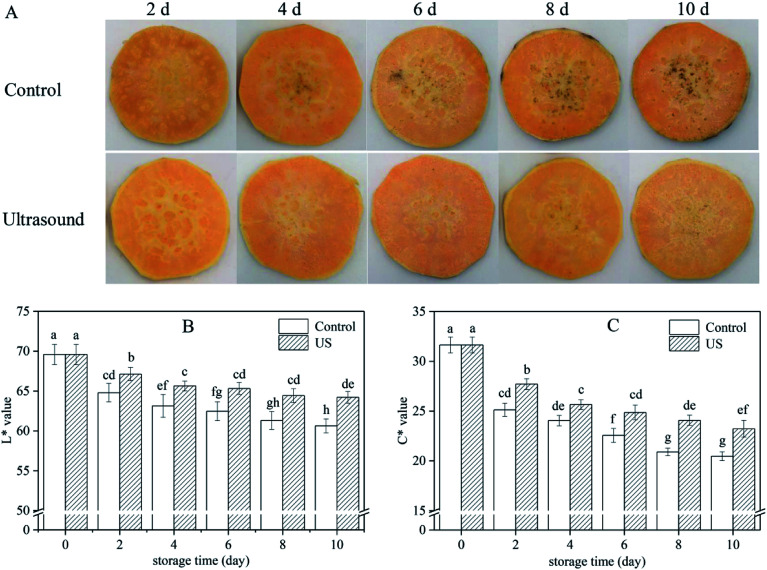
Effect of ultrasound treatment on appearance (A), L* (B) and C* (C) of fresh-cut sweet potato slices during 10 d of storage at 4 °C (control: slices treated in distilled water for 10 min; US: slices treated in ultrasound bath at 40 kHz frequency and 100 W power density for 10 min). Values are the means ± SD (*n* = 3). Different letters indicate a significant difference (*p* < 0.05).

Browning is one of the main factors restricting the quality and shelf-life of fresh-cut sweet potato. US-treated sweet potato slices had the lower surface browning in comparison to the control (Fig. 1A). L* parameter is the best indicator for assessing the appearance of browning while C* parameter represents the saturation degree which is proportional to color intensity.^39^ Color changes in sweet potato slices indicated by L* and C* were influenced by both US treatment and cold storage, and a continual reduction was observed when storage time was extended, in agreement with the appearance of images in Fig. 1. Reductions in luminosity and chroma may be related to cell rupture due to minimal cutting. However, compared with the control, US treatment significantly (*p* < 0.05) inhibited the decrease of L* (*p* = 0.000003) and C* (*p* = 0.000018) values throughout the entire storage phase. For instance, L* value of the sweet potato slices treated with US was 64.22 on the 10th day, that is 5.6% higher than the control (Fig. 1B) and C* value treated with US was 23.23, 11.9% higher than the control (Fig. 1C). These findings suggested that US treatment was conducive to maintaining the surface color of fresh-cut sweet potato during cold storage. Similar results have been reported whereby US treatment efficiently inhibited browning in other fresh-cut products.^25,40^ In contrast, Santos *et al.*^7^ observed a decline in color parameters of fresh-cut mango in response to US. These different evolutions in color may be relevant to fruit varieties, ultrasonic power and processing time.^23^

### 3.2. Total phenolic content and PPO, POD, PAL activities

**Fig. 2:**
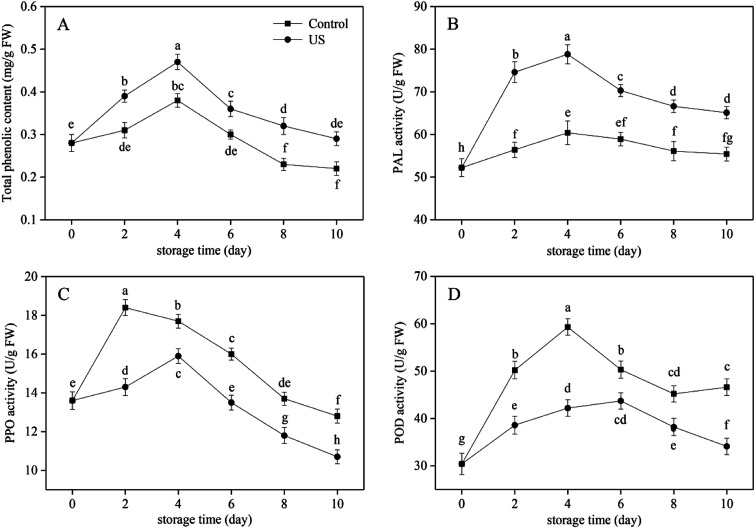
Effect of ultrasound treatment on total phenolic content (A), PAL (B), PPO (C) and POD (D) activity of fresh-cut sweet potato slices during 10 d of storage at 4 °C. Values are the means ± SD (*n* = 3). Different letters indicate a significant difference (*p* < 0.05).

Phenolic substances in sweet potato have important antioxidant activities in chelating active metal ions and inhibiting lipid peroxidation.^41^ They participate in the enzymatic browning reaction as oxidation substrates of phenolase. The total phenolic content in the control and US-treated sweet potato slices all progressively increased in the first 4 days and then decreased with prolonged storage (Fig. 2A), and US-treated root slices possessed significantly higher content than that of the control (*p* < 0.05; *p* = 0.000037). Regression analysis showed that the changes in total phenolic content were positively correlated with PAL activity in Fig. 2B (*r* = 0.818, *p* < 0.01; *p* = 6.1752 × 10^−9^). The increase in total phenolic content during 0–4 d of storage may be caused by stimulated PAL activity following mechanical damage from peeling and cutting. This can stimulate the synthesis of phenolic substances in the phenylpropanoid metabolic pathway to repair damaged tissues.^42^ The later decline may be attributed to the continuous consumption by oxidative browning and the delay in the synthesis of phenols by decreased PAL activity (Fig. 2B). At the end of the storage phase, the content of total phenols in the control group declined significantly by 21.4% compared with the initial value. US treatment was more beneficial to the retention of total phenolic content in fresh-cut sweet potato, which was 24.1% higher than the control at day 10. Similarly, Lagnika *et al.*^27^ demonstrated that the phenolic content in white mushroom treated with US for 10 min was 37.5% higher than in the control stored at 4 °C.

As the crucial and rate-limiting enzyme in the first committed step of phenylpropanoid pathway involving in the synthesis of phenolic defense substances, PAL activity in fresh-cut sweet potato was observed to be induced due to the stress response caused by mechanical wounding in the initial 4 days of cold storage and then decreased due to aging and browning consumption till the end. This was consistent with the overall development trend of total phenolic content (Fig. 2A and B). The activation of PAL activity subjected to minimal processing was also found in carrot slices and shreds.^43^ Treatment with US for 10 min contributed to a significant increase in PAL activity in comparison to the control during the entire refrigeration period (*p* < 0.05; *p* = 0.000005). The peak level of PAL activity in US was 78.8 U g^−1^ FW, representing an increase of 23.4% compared with the control (Fig. 2B). Previous research suggested that the application of US operated at 350 W for 10 min resulted in a higher PAL activity in peach fruit.^44^ These results may be attributed to the fact that free radicals produced by sonolysis of water put sweet potato tissues under oxidative stress and further improved PAL activity. In effect it enhanced the ability of cells to resist external oxidative damage.^28^ Therefore, the PAL activity and total phenolic content of US-treated sweet potato slices were induced at higher levels under the combined action of mechanical and oxidative stress injury during refrigerated storage.

US treatment has both activation and passivation effects on physiological enzyme activity and is mainly associated with the power intensity, processing time and temperature. Kentish and Feng^22^ have suggested that the inactivation effect of US depends on the chemical structure of proteins and the tolerance of different enzymes to US. Changes in PPO and POD activities in fresh-cut sweet potato are illustrated in Fig. 2C and D, and both of them revealed an earlier increase and later decrease trend along with storage time. Compared with the control group, US treatment for 10 min significantly inhibited enzyme activities (*p* < 0.05; *p* = 9.8373 × 10^−7^ and *p* = 2.5924 × 10^−8^, respectively). Among them, peak time of PPO was postponed from day 2 to day 4 and peak time of POD was postponed from day 4 to day 6 in response to US treatment. Moreover, PPO and POD peak levels of US-treated sweet potato slices were 15.9 and 43.7 U g^−1^ FW; thus, lowered by 13.6% and 26.3% compared with the peak level of untreated slices, respectively. It has been demonstrated that PPO and POD activities of tomato extract and fresh-cut potato were also suppressed by US treatment,^23,45^ which supported our results.

Overall, the application of US treatment operating at 40 kHz for 10 min effectively increased PAL activity and total phenolic content, yet decreased PPO and POD activities in fresh-cut sweet potato slices. These findings suggested that the inhibitory effect of US on browning of sweet potato slices did not result from the reduction of phenolic substrates. Other studies on fresh-cut potato also revealed that: firstly, browning development was only partially correlated with the PAL activity at the early stage of mechanical wounding; and secondly, phenolic substrate concentration was not rate-limiting in tissue browning.^10^ It was therefore suggested that the browning inhibition of fresh-cut sweet potato following US treatment was exerted by reducing the activity of PPO and POD, which were typical enzymes known to be involved in browning.

### 3.3. Oxidative stress and antioxidant enzymes

**Fig. 3:**
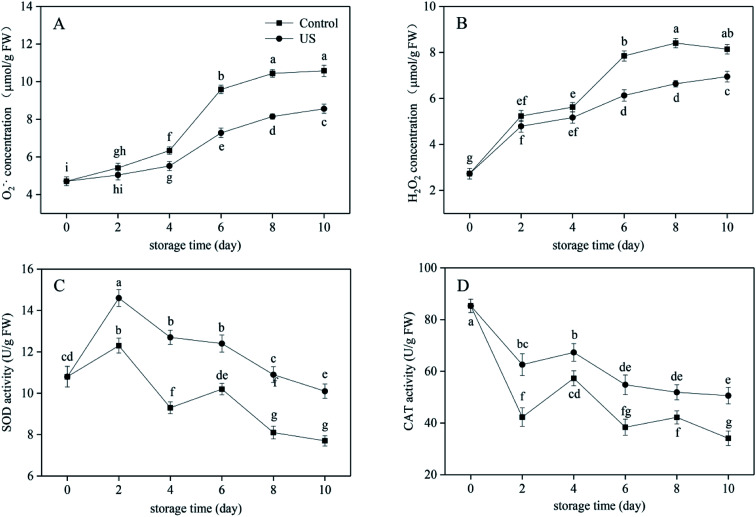
Effect of ultrasound treatment on O_2_^−^˙ (A) and H_2_O_2_ (B) concentration, SOD (C) and CAT (D) activity of fresh-cut sweet potato slices during 10 d of storage at 4 °C. Values are the means ± SD (*n* = 3). Different letters indicate a significant difference (*p* < 0.05).

The mechanical injury caused by fresh-cutting triggers an imbalance in reactive oxygen metabolism and causes the accumulation of reactive oxygen species such as O_2_^−^˙ and H_2_O_2_ in sweet potato tissues. This accelerates the peroxidation reaction of membrane lipids, resulting in the oxidative damage being done to plant cells.^13,46^ As shown in Fig. 3A and B, O_2_^−^˙ concentration in fresh-cut sweet potato generally increased gradually within the whole cold storage stage while H_2_O_2_ concentration increased in the first 8 days but then declined till the end. There was no significant difference in O_2_^−^˙ concentration between the US treatment and the control for the first 2 days, and no significant difference in H_2_O_2_ concentration in the first 4 days (*p* > 0.05; *p* = 0.058). Sweet potato slices treated with US for 10 min significantly lowered O_2_^−^˙ and H_2_O_2_ concentrations during the subsequent storage period, respectively (*p* < 0.05; *p* = 3.8046 × 10^−10^ and *p* = 0.000013, respectively). As for O_2_^−^˙, concentration in the US group was 8.56 μmol g^−1^ FW at the end of storage, which represented a reduction by 19.1% compared with the control group. As for H_2_O_2_, concentrations in US group were 6.64 μmol g^−1^ FW at day 8 and 6.95 μmol g^−1^ FW at day 10, which represents reductions by 21.0% and 14.6% compared with the control group, respectively. Therefore, application of US processing effectively alleviated the oxidative stress in sweet potato slices stored at 4 °C.

As important ROS scavenging enzymes in plant cells, SOD can specifically detoxify O_2_^−^˙ by forming H_2_O_2_ and CAT can decompose H_2_O_2_ into molecular water and oxygen, thus preventing ROS causing damage to the cell membrane.^47,48^ According to Fig. 3C, SOD activity in US-treated sweet potato slices first increased and then progressively decreased while SOD activity in the control indicated a “up-down-up-down” trend. They both reached the peak level (14.6 U g^−1^ FW in the US treatment and 12.3 U g^−1^ FW in the control) at day 2. This may have been due to cutting injury coupled with cold storage inducing the rapid increase of SOD activity in order to reduce the damage caused by free radicals. US-treated slices maintained significantly higher SOD levels in comparison to the control during the entire refrigeration period (*p* < 0.05), thus retarding the oxidation process of fresh-cut sweet potato. Meanwhile, Fig. 3D showed that the increase in CAT activity was induced by accumulated H_2_O_2_ as tissue and cell damage after fresh cutting needed some time. Therefore, CAT activities in slices treated with US and the control increased to different degrees from the 2nd day, and then decreased after rising to a certain extent on the 4th day. Overall, the more severe the adversity stress is, the more obvious the change in stress response of CAT activity is. Treatment with US significantly increased CAT activity in sweet potato slices than in the control (*p* < 0.05; *p* = 0.000004). At day 10, CAT activity of slices in the US group was 1.48 times higher than in the control group.

Cutting injury can induce the accumulation of ROS including O_2_^−^˙ and H_2_O_2_, leading to changes in protective enzymes including SOD and CAT that mainly respond to oxidative stress. There was little information on the free radical metabolism and antioxidation system subjected to US available in the postharvest context. In the current study, US treatment operating at 40 kHz for 10 min significantly reduced O_2_^−^˙ and H_2_O_2_ concentrations at different periods. Also, US effectively inhibited the decline in SOD and CAT activity, thus retaining the reactive oxygen scavenging enzyme system at a high level. Moreover, Yang *et al.* reported that ultrasound at 40 kHz for 10 min combined with salicylic acid induced higher activities of SOD and CAT, specifically protecting peach fruit against oxidative damage compared to control or salicylic acid treatment alone.^49^ However, the results differed from those documented by Santos *et al.*^7^ for fresh-cut mangoes, where the activities of SOD and CAT were not stimulated by sonication. Thus, various fruit tissues respond differently to ultrasonic treatment.

### 3.4. Changes in total antioxidant capacity

**Fig. 4:**
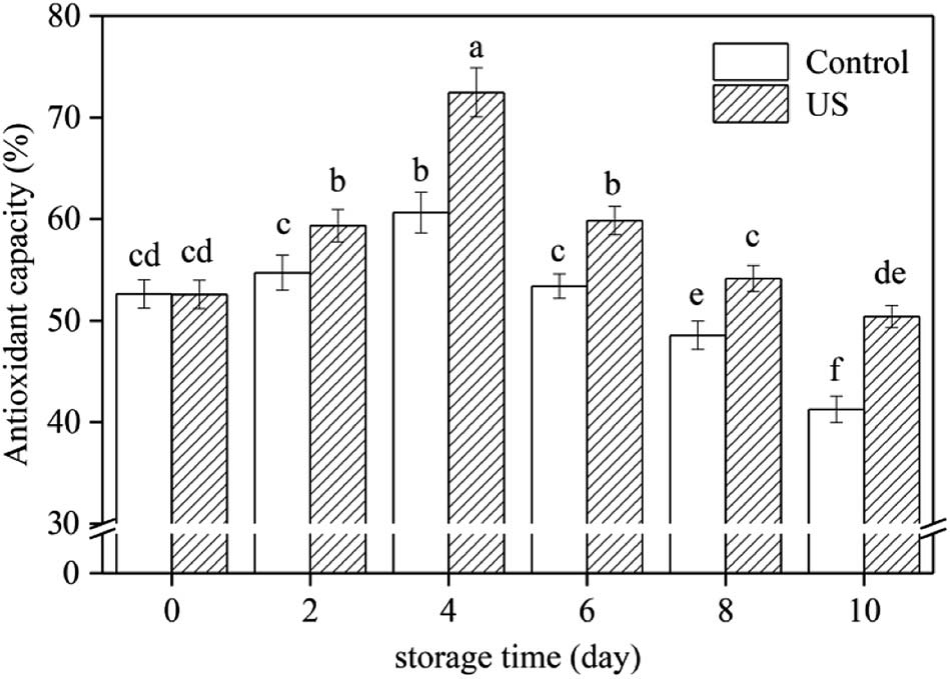
Effect of ultrasound treatment on antioxidant capacity of fresh-cut sweet potato slices as determined by DPPH assay during 10 d of storage at 4 °C. Values are the means ± SD (*n* = 3). Different letters indicate a significant difference (*p* < 0.05).

The scavenging mechanisms of free radicals by antioxidant substances are mainly achieved by hydrogen atom transfer and single electron transfer.^50^ DPPH radical neutralization by direct reduction or quenching through hydrogen atom or single electron transfer, can estimate the total antioxidant capacity of fresh-cut sweet potato slices.^28,50^ The interaction of US treatment and storage time had a significant effect on the antioxidant activity in root slices (Fig. 4). Antioxidant capacity in root slices increased during the first 4 days and then decreased later, which was like the change in total phenolic content (Fig. 2A). Regression analysis showed that the changes in antioxidant capacity were positively correlated with total phenolic content (*r* = 0.954, *p* < 0.01). Therefore, polyphenols are the main antioxidant substances in sweet potato roots.

In this study, treatment with US for 10 min significantly improved antioxidant capacity compared to the control under refrigerated storage conditions (*p* < 0.05; *p* = 3.5942 × 10^−7^). Following 4 d and 10 d of storage, antioxidant capacities in US-treated slices were 16.3% and 18.2% higher than in untreated slices, respectively. This may be due to the larger amount of total phenols in sweet potato slices subjected to ultrasonic treatment.^28^ Inducing a high level of antioxidant capacity was also observed in mushroom exposed to US treatment for 10 min.^27^ These results support the hypothesis suggested by Cisneros-Zevallos,^51^ in which controlled abiotic stress processing may be used to promote the accumulation of phytochemicals known to be beneficial to plants.

**Fig. 5:**
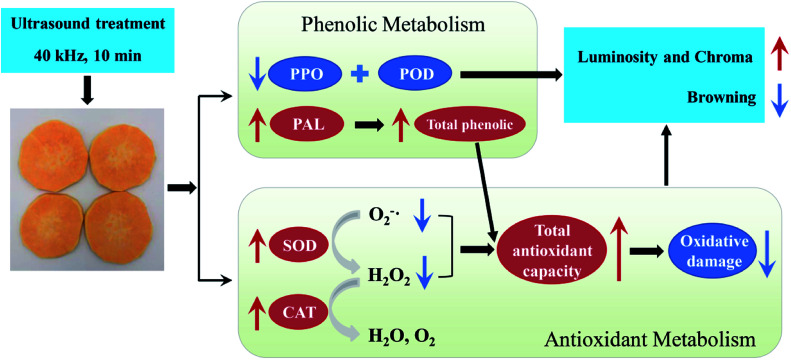
A proposed model for the role of US inhibiting browning of fresh-cut sweet potato. US treatment reduced the activity of PPO and POD to delay enzymatic browning. On the other hand, US increased total antioxidant capacity to alleviate oxidative stress. They both inhibited the browning of sweet potato slices. The up arrow (red) represents promotion, down arrow (blue) represents inhibition.

As reported by Sun *et al.*,^48^ improving the antioxidant capacity to mitigate oxidative damage was directly related to successful browning inhibition in fresh-cut lotus root. In the present study, application of US preserved higher total phenolic content and antioxidant capacity levels, thus accelerating free radical metabolism and further alleviating oxidative damage to fresh-cut sweet potato. This should be another explanation for ultrasound preventing browning of sweet potato slices. Finally, combined the results in Fig. 1–4, a proposed model for the role of US inhibiting browning of fresh-cut sweet potato was showed in Fig. 5.

## 4. Conclusions

Different enzymes have different levels of tolerance to ultrasonic inactivation due to various chemical structure of proteins. In this study, US treatment effectively reduced PPO and POD activities in fresh-cut sweet potato stored at 4 °C, thereby delaying the enzymatic reaction with phenolic substances and preventing browning. Conversely, PAL activity subjected to US treatment was significantly higher than that of the control, thus maintaining a higher content of total phenol. As well, the formation of free radicals by US produced oxidative stress on sweet potato slices. Consequently, antioxidant enzymes including SOD and CAT increased in US-treated slices to suppress the accumulations of O_2_^−^˙ and H_2_O_2_. It was furthermore demonstrated that US treatment enhanced antioxidant capacity in slices by maintaining more total phenolic content and inducing SOD and CAT activities, which helped to alleviate the oxidative damage caused by ROS metabolism.

In conclusion, treatment with ultrasound at 40 kHz for 10 min was conducive to maintaining the surface color and inhibiting browning of fresh-cut sweet potato during cold storage at 4 °C. The inhibition was realized by two processes: (1) reducing the activity of PPO and POD so that enzymatic browning was delayed; and (2) improving antioxidant capacity to combat oxidative stress.

## Conflicts of interest

There are no conflicts to declare.

## Supplementary Material

## References

[cit1] Huang C. L., Liao W. C., Chan C. F., Lai Y. C. (2014). Storage performance of Taiwanese sweet potato cultivars. J. Food Sci. Technol..

[cit2] Philpott M., Gould K. S., Markham K. R., Lewthwaite S. L., Ferguson L. R. (2003). Enhanced coloration reveals high antioxidant potential in new sweetpotato cultivars. J. Sci. Food Agric..

[cit3] Bovell-Benjamin A. C. (2007). Sweet Potato: a review of its past, present, and future role in human nutrition. Adv. Food Nutr. Res..

[cit4] Qi H. P., Hu W. Z., Jiang A. L., Tian M. X., Li Y. Q. (2011). Extending shelf life of fresh-cut ‘Fuji’ apples with chitosan-coatings. Innovative Food Sci. Emerging Technol..

[cit5] Harich M., Maherani B., Salmieri S., Lacroix M. (2018). Evaluation of antibacterial activity of two natural bio-preservatives formulations on freshness and sensory quality of ready to eat (RTE) foods. Food Control.

[cit6] Francis G. A., Gallone A., Nychas G. J., Sofos J. N., Colelli G., Amodio M. L., Spano G. (2012). Factors Affecting Quality and Safety of Fresh-Cut Produce. Crit. Rev. Food Sci. Nutr..

[cit7] Santos J. G., Fernandes F. A. N., Oliveira L. S., Miranda M. R. A. (2015). Influence of Ultrasound on Fresh-Cut Mango Quality Through Evaluation of Enzymatic and Oxidative Metabolism. Food Bioprocess Technol..

[cit8] Galindo-Pérez M. J., Quintanar-Guerrero D., Mercado-Silva E., Real-Sandoval S. A., Zambrano-Zaragoza M. L. (2015). The Effects of Tocopherol Nanocapsules/Xanthan Gum Coatings on the Preservation of Fresh-Cut Apples: Evaluation of Phenol Metabolism. Food Bioprocess Technol..

[cit9] Oms-Oliu G., Rojas-Graü M. A., González L. A., Varela P., Soliva-Fortuny R., Hernando M. I. H., Munuera I. P., Fiszman S., Martín-Belloso O. (2010). Recent approaches using chemical treatments to preserve quality of fresh-cut fruit: a review. Postharvest Biol. Technol..

[cit10] Cantos E., Tudela J. A., Gil M. I., Espín J. C. (2003). Phenolic Compounds and Related Enzymes Are Not Rate-Limiting in Browning Development of Fresh-Cut Potatoes. J. Agric. Food Chem..

[cit11] Thomas V. (2010). Phenylpropanoid Biosynthesis. Mol. Plant.

[cit12] Saltveit M. E. (2000). Wound induced changes in phenolic metabolism and tissue browning are altered by heat shock. Postharvest Biol. Technol..

[cit13] Lin Y. F., Lin H. T., Lin Y. X., Zhang S., Chen Y. H., Jiang X. J. (2016). The roles of metabolism of membrane lipids and phenolics in hydrogen peroxide-induced pericarp browning of harvested longan fruit. Postharvest Biol. Technol..

[cit14] Aguayo E., Requejo-Jackman C., Stanley R., Woolf A. (2010). Effects of calcium ascorbate treatments and storage atmosphere on antioxidant activity and quality of fresh-cut apple slices. Postharvest Biol. Technol..

[cit15] Toivonen P. M. A., Brummell D. A. (2008). Biochemical bases of appearance and texture changes in fresh-cut fruit and vegetables. Postharvest Biol. Technol..

[cit16] Waimaleongora-Ek P., Corredor A. J. H., No H. K., Prinyawiwatkul W., King J. M., Janes M. E., Sathivel S. (2008). Selected Quality Characteristics of Fresh-Cut Sweet Potatoes Coated with Chitosan during 17-Day Refrigerated Storage. J. Food Sci..

[cit17] Sgroppo S. C., Vergara L. E., Tenev M. D. (2010). Effects of sodium metabisulphite and citric acid on the shelf life of fresh cut sweet potatoes. Span. J. Agric. Res..

[cit18] Calder B. L., Kash E. A., Davis-Dentici K., Bushway A. A. (2011). Comparison of Sodium Acid Sulfate to Citric Acid to Inhibit Browning of Fresh-Cut Potatoes. J. Food Sci..

[cit19] Awad T. S., Moharram H. A., Shaltout O. E., Asker D., Youssef M. M. (2012). Applications of ultrasound in analysis, processing and quality control of food: a review. Food Res. Int..

[cit20] Chandrapala J., Oliver C., Kentish S., Ashokkumar M. (2012). Ultrasonics in food processing. Ultrason. Sonochem..

[cit21] O’Donnell C. P., Tiwari B. K., Bourke P., Cullen P. J. (2010). Effect of ultrasonic processing on food enzymes of industrial importance. Trends Food Sci. Technol..

[cit22] Kentish S., Feng H. (2014). Applications of Power Ultrasound in Food Processing. Annu. Rev. Food Sci. Technol..

[cit23] Amaral R. D. A., Benedetti B. C., Pujola M., Achaerandio I., Bachelli M. L. B. (2015). Effect of Ultrasound on Quality of Fresh-Cut Potatoes During Refrigerated Storage. Food Eng. Rev..

[cit24] Birmpa A., Sfika V., Vantarakis A. (2013). Ultraviolet light and ultrasound as non-thermal treatments for the inactivation of microorganisms in fresh ready-to-eat foods. Int. J. Food Microbiol..

[cit25] Jang J. H., Moon K. D. (2011). Inhibition of polyphenol oxidase and peroxidase activities on fresh-cut apple by simultaneous treatment of ultrasound and ascorbic acid. Food Chem..

[cit26] Fan K., Zhang M., Jiang F. J. (2019). Ultrasound Treatment to Modified Atmospheric Packaged Fresh-cut Cucumber: Influence on Microbial Inhibition and Storage Quality. Ultrason. Sonochem..

[cit27] Lagnika C., Zhang M., Mothibe K. J., Bashari M. (2013). Effects of ultrasound and high pressure argon on physico-chemical properties of white mushrooms (*Agaricus bisporus*) during postharvest storage. Postharvest Biol. Technol..

[cit28] Yeoh W. K., Ali A. (2017). Ultrasound treatment on phenolic metabolism and antioxidant capacity of fresh-cut pineapple during cold storage. Food Chem..

[cit29] Ordóñez-Santos L. E., Martínez-Girón J., Arias-Jaramillo M. E. (2017). Effect of ultrasound treatment on visual color, vitamin C, total phenols, and carotenoids content in Cape gooseberry juice. Food Chem..

[cit30] Liu X., Lu Y. Z., Yang Q., Yang H. Y., Li Y., Zhou B. Y., Li T. T., Guo Y. (2018). Cod peptides inhibit browning in fresh-cut potato slices: a potential anti-browning agent of random peptides for regulating food properties. Postharvest Biol. Technol..

[cit31] Zhou P., Smith N. L., Chang Y. L. (1993). Potential purification and some properties of Monroe apple peel polyphenol oxidase. J. Agric. Food Chem..

[cit32] Xu W. T., Peng X. L., Luo Y. B., Wang J. A., Guo X., Huang K. L. (2009). Physiological and biochemical responses of grapefruit seed extract dip on 'Redglobe' grape. LWT--Food Sci. Technol..

[cit33] Assis J. S., Maldonado R., Munoz T., Escribano M. I., Merodio C. (2001). Effect of high carbon dioxide concentration on PAL activity and phenolic contents in ripening cherimoya fruit. Postharvest Biol. Technol..

[cit34] Xu M. J., Dong J. F., Zhang M., Xu X. B., Sun L. N. (2012). Cold-induced endogenous nitric oxide generation plays a role in chilling tolerance of loquat fruit during postharvest storage. Postharvest Biol. Technol..

[cit35] Patterson B. D., Macrae E. A., Ferguson I. B. (1984). Estimation of hydrogen peroxide in plant extracts using titanium (IV). Anal. Biochem..

[cit36] Vicente A. R., Martínez G. A., Chaves A. R., Civello P. M. (2006). Effect of heat treatment on strawberry fruit damage and oxidative metabolism during storage. Postharvest Biol. Technol..

[cit37] Bassal M., El-Hamahmy M. (2011). Hot water dip and preconditioning treatments to reduce chilling injury and maintain postharvest quality of Navel and Valencia oranges during cold quarantine. Postharvest Biol. Technol..

[cit38] Brand-Williams W., Cuvelier M. E., Berset C. (1995). Use of free radical method to evaluate antioxidant activity. LWT--Food Sci. Technol..

[cit39] Bico S. L. S., Raposo M. F. J., Morais R. M. S. C., Morais A. M. M. B. (2009). Combined effects of chemical dip and/or carrageenan coating and/or controlled atmosphere on quality of fresh-cut banana. Food Control.

[cit40] Chemat F., Huma Z., Khan M. K. (2010). Applications of Ultrasound in Food Technology: Processing, Preservation and Extraction. Ultrason. Sonochem..

[cit41] Oliveira A. C. D., Valentim I. B., Silva C. A., Bechara E. J. H., Barros M. P., Mano C. M., Goulart M. O. F. (2009). Total phenolic content and free radical scavenging activities of methanolic extract powders of tropical fruit residues. Food Chem..

[cit42] Wang Q. G., Cao Y., Zhou L. L., Jiang C. Z., Feng Y. Y., Wei S. D. (2015). Effects of Postharvest Curing Treatment on Flesh Colour and Phenolic Metabolism in Fresh-cut Potato Products. Food Chem..

[cit43] Heredia J. B., Cisneros-Zevallos L. (2009). The effect of exogenous ethylene and methyl jasmonate on pal activity, phenolic profiles and antioxidant capacity of carrots (*Daucus carota*) under different wounding intensities. Postharvest Biol. Technol..

[cit44] Yang Z. F., Cao S. F., Cai Y. T., Zheng Y. H. (2011). Combination of salicylic acid and ultrasound to control postharvest blue mold caused by *Penicillium expansum* in peach fruit. Innovative Food Sci. Emerging Technol..

[cit45] Ercan S. S., Soysal C. (2011). Effect of ultrasound and temperature on tomato peroxidase. Ultrason. Sonochem..

[cit46] Apel K., Hirt H. (2004). Reactive Oxygen Species: Metabolism, Oxidative Stress, and Signal Transduction. Annu. Rev. Plant Biol..

[cit47] Bolwell G. P., Wojtaszek P. (1997). Mechanisms for the generation of reactive oxygen species in plant defence response. Physiol. Mol. Plant Pathol..

[cit48] Sun Y., Zhang W., Zeng T., Nie Q. X., Zhang F. Y., Zhu L. Q. (2015). Hydrogen sulfide inhibits enzymatic browning of fresh-cut lotus root slices by regulating phenolic metabolism. Food Chem..

[cit49] Yang Z. F., Cao S. F., Zheng Y. H., Jiang Y. M. (2012). Combined Salicyclic Acid and Ultrasound Treatments for Reducing the Chilling Injury on Peach Fruit. J. Agric. Food Chem..

[cit50] Prior R. L., Wu X., Schaich K. (2005). Standardized Methods for the Determination of Antioxidant Capacity and Phenolics in Foods and Dietary Supplements. J. Agric. Food Chem..

[cit51] Cisneros-Zevallos L. (2003). The Use of Controlled Postharvest Abiotic Stresses as a Tool for Enhancing the Nutraceutical Content and Adding-Value of Fresh Fruits and Vegetables. J. Food Sci..

